# Tumour-on-Chip Models for the Study of Ovarian Cancer: Current Challenges and Future Prospects

**DOI:** 10.3390/cancers17193239

**Published:** 2025-10-06

**Authors:** Sung Yeon Lim, Lamia Sabry Aboelnasr, Mona El-Bahrawy

**Affiliations:** 1 Department of Metabolism, Digestion and Reproduction, Imperial College, London W12 0NN, UK; sung.lim22@imperial.ac.uk (S.Y.L.); l.aboelnasr22@imperial.ac.uk (L.S.A.); 2 Department of Pathology, Faculty of Medicine, Menoufia University, Shibin el Kom 6131567, Egypt; 3 Department of Pathology, Faculty of Medicine, University of Alexandria, Bab Sharqi 5424041, Egypt

**Keywords:** ovarian cancer, tumour-on-chip, microfluidics, tumour microenvironment, personalized medicine

## Abstract

**Simple Summary:**

Ovarian cancer is one of the most lethal cancers in women, largely because it is often detected late, shows high diversity with intra tumour and inter tumour heterogeneity, and frequently becomes resistant to treatment. Current laboratory and animal models do not fully capture the complexity of ovarian tumours, making it difficult to predict how the disease develops or how patients will respond to therapies. Tumour-on-chip technology is an emerging tool that uses microfluidic devices to recreate key features of human tumours. In this review, we summarise how these systems have been applied to ovarian cancer research, highlight their advantages and limitations, and propose strategies for building more reliable and patient-relevant models. By considering future development of this technology, we aim to accelerate discoveries in ovarian cancer biology and improve prospects for personalised treatment.

**Abstract:**

Ovarian cancer is a highly lethal malignancy, characterised by late-stage diagnosis, marked inter- and intra-tumoural heterogeneity, and frequent development of chemoresistance. Existing preclinical models, including conventional two-dimensional cultures, three-dimensional spheroids, and organoids, only partially recapitulate the structural and functional complexity of the ovarian tumour microenvironment (TME). Tumour-on-chip (CoC) technology has emerged as a promising alternative, enabling the co-culture of tumour and stromal cells within a microengineered platform that incorporates relevant extracellular matrix components, biochemical gradients, and biomechanical cues under precisely controlled microfluidic conditions. This review provides a comprehensive overview of CoC technology relevant to ovarian cancer research, outlining fabrication strategies, device architectures, and TME-integration approaches. We systematically analyse published ovarian cancer-specific CoC models, revealing a surprisingly limited number of studies and a lack of standardisation across design parameters, materials, and outcome measures. Based on these findings, we identify critical technical and biological considerations to inform the rational design of next-generation CoC platforms, with the aim of improving their reproducibility, translational value, and potential for personalised medicine applications.

## 1. Introduction

Ovarian cancer (OC) is the 8th leading cause of cancer mortality in women [1]. It is a diverse collection of diseases divided into five distinct histological subtypes. The majority (~90%) of these subtypes fall under epithelial ovarian cancer, which includes serous, endometrioid, clear-cell, and mucinous carcinomas [2]. Of note, high-grade serous carcinoma (HGSC) is the most lethal and frequently diagnosed histotype and is characterised by ubiquitous TP53 mutation and extreme chromosomal instability [3,4]. Non-epithelial ovarian cancers are rare and include germ-cell and sex cord stromal tumours. All histotypes are complex and extremely heterogeneous, with a lack of early detection strategies and frequent development of chemotherapy resistance [2].

Accurate and representative preclinical models for OC are crucial to deconvolute the disease’s complexity, improve therapeutic strategies, and ultimately enhance patient outcomes. In vitro models currently include 2D cell lines, 3D spheroids, and organoids. Established 2D cancer cell lines serve as an affordable and easily reproducible option for modelling OC. However, they lack 3D structure and the human TME. There is also increasing evidence about the mislabelling, cross-contamination, and secondary genomic alterations that reduce the reliability of these models [5,6]. Spheroids and organoids offer the advantage of preserving aspects of the 3D microstructure and the TME [7,8]. Spheroids are formed from the self-aggregation of cells and are reported to morphologically resemble OC aggregates in patient ascites [9]. Organoids have added 3D complexity being derived from stem cells, and patient-derived organoids have recently shown promise in reflecting both morphological phenotypes and genetic heterogeneity of their parent tumours [10]. These 3D models may preserve some cell–cell and cell–extracellular matrix (ECM) interactions (especially when used with ECM-mimicking scaffolds or gels) and generate nutrient and pharmacological gradients otherwise unobtainable with 2D cells [11]. However, they lack more complex TME interactions such as vascularisation or physiological (flow or mechanical) cues [9].

In vivo models have been an important tool for studying OC. In xenograft models, patient tumour tissues are injected into immunocompromised or humanised mice, closely modelling patient tumours in an in vivo environment [12]. Depending on the location and type of injection, different stages of OC can be modelled, making this an essential system for studying drug response and disease development [13]. Syngeneic models use tumour cells from the same genetic background as the host, allowing researchers to study tumour–immune system interactions in immunocompetent systems [10,14]. Genetically Engineered Mouse Models (GEMMs) introduce human-relevant mutations into mice to better replicate specific subtypes of ovarian cancer [15]. Nevertheless, in vivo models have important limitations, including animal expenses, inter-species differences, ethical considerations, and low throughput [6].

Tumour-on-a-chip (cancer-on-chip, CoC) models use organ-on-a-chip technology to construct tissue models within a microfluidics-controlled micro/nano environment [16,17]. Cancer cells are cultured with relevant TME cells and ECM components in one or more microfluidic channels to emulate the intricate dynamics within the TME. Media can be perfused through the microchannels which adds important biomechanical cues, such as fluid shear stress, to further replicate the environment in vivo [18]. There is also potential for ex vivo models culturing patient-derived explants (tissue and organ slices) within a microfluidic chip system [19]. CoC models aim to provide more representative, adaptable, and cost-effective methods for characterising disease development and drug efficacy. Moreover, they have high translational potential for personalised screening of therapies using patient biopsies, which could significantly enhance precision medicine approaches. Despite their promise, CoC models, especially in OC, are not yet widely adopted in research, clinical trials, and pharmaceutical industries [20].

This report provides a literature review on the current state of CoC technology and its application in OC research. CoC designs are categorised into specific subtypes, with an analysis of their respective strengths and limitations. Previously published CoC models for OC are identified, revealing common design aspects and specific challenges that may have hindered the widespread implementation of this technology in OC research. Drawing on these insights, we outline a series of design decision steps and parameters intended to guide the systematic development of future CoC platforms. These considerations aim to improve the standardisation, reproducibility, and translational relevance of OC-specific CoC models.

## 2. Materials and Methods

### 2.1. Literature Review Strategy

A structured literature review was performed to identify tumour-on-a-chip (CoC) models applied to ovarian cancer (OC). Searches were conducted in PubMed, Scopus, and Web of Science up to 13 February 2025. A combination of Medical Subject Headings (MeSH) and free-text keywords was used, with OC and CoC concepts combined using the Boolean operator AND. Search terms for OC included “Ovarian Neoplasms” [MeSH], “ovarian cancer”, “ovarian carcinoma”, and “ovarian tumour/tumor”. Terms related to CoC models included “cancer-on-chip”, “tumor-on-chip/tumour-on-chip”, “organ-on-chip”, “microfluidic cancer model”, “microfluidic tumor model”, and “microfluidics AND cancer”.

### 2.2. Study Selection Criteria

Articles were screened based on the following inclusion criteria:

Original experimental studies focused on the use of microfluidic or organ-on-chip systems for modelling ovarian cancer.

Use of established OC cell lines or patient-derived cells.

Relevance to TME modelling.

Exclusion criteria included:

Review articles.

Studies focused solely on diagnostic, analytical, or biosensing microfluidic approaches without TME modelling.

Articles unrelated to ovarian cancer.

All eligible studies were screened manually by two independent reviewers. Discrepancies were resolved through consensus discussion.

### 2.3. Data Extraction

The following data were extracted from the included studies:

Study objective and experimental focus

Design and fabrication approach of the chip

OC cell types used, including TME components (e.g., fibroblasts, endothelial, immune cells)

Scaffold or extracellular matrix (ECM) materials

Output measurements (e.g., invasion, drug response, proliferation)

Major findings and conclusions

### 2.4. Design Considerations

From the reviewed literature, key design considerations and parameters for developing CoC models in OC were identified. These include the choice of fabrication technique, selection of biomaterials, integration of relevant TME components, control of microfluidic flow parameters, and compatibility with analytical readouts. These considerations are summarised to provide a framework for researchers aiming to create reproducible, physiologically relevant models.

## 3. Results

### 3.1. Review of CoC Manufacturing

The key component of a CoC model is its microfluidic system, which must be biocompatible and contain the microstructures and microchannels for arranging cells and controlling fluid flow. These intricate designs on biocompatible polymers are often produced using replica moulding techniques involving photo- and soft-lithography.

**Figure 1:**
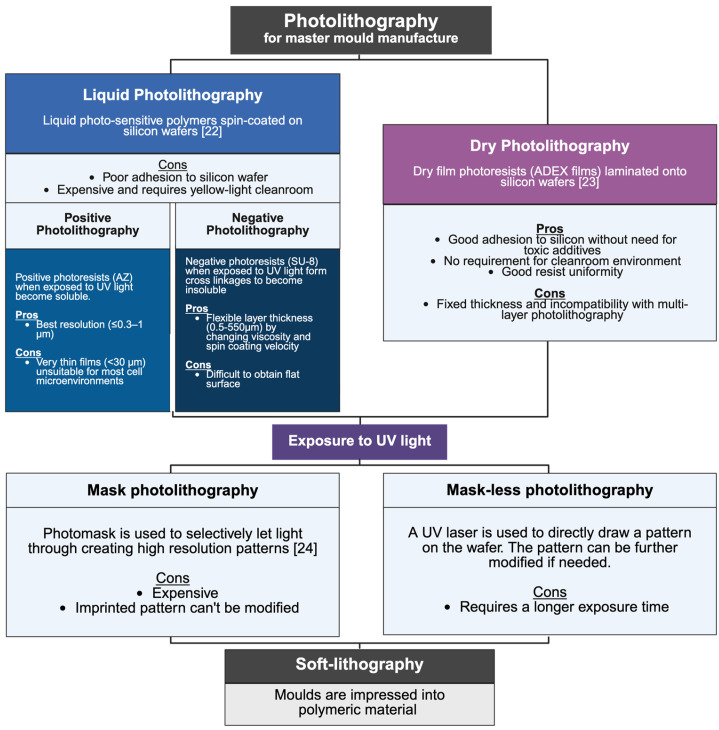
Photo- and soft-lithography are often used to manufacture CoC models, and the figure summarises the process from mould fabrication to material impression, starting at the top of the figure. To create the master mould, photolithography can either use liquid polymers or dry film photoresists. Selective exposure to UV light can be done with either a photomask or a laser. The completed mould is used to shape polymeric material into the desired 3D microstructure [22,23,24]. Created in BioRender. Lim, P. (2025) https://BioRender.com/h6uke0k, accessed on 20 August 2025.

Photolithography involves selectively exposing photosensitive materials (photoresists) to UV light to produce a patterned mould [21]. A silicon wafer is coated with a photoresist material and exposed to UV light using either a photomask or direct laser writing. Two main approaches are used: liquid photolithography, which commonly employs negative resists such as SU-8, and dry film photolithography, a more recently developed alternative that laminates prefabricated films onto wafers [21,22,23]. Regardless of the approach, the resulting master mould is used for soft-lithography, where the micropattern is transferred onto polymeric materials. The key steps, variations, and advantages of these techniques are summarised in Figure 1 [22,23,24].

The most common material used for soft lithography is polydimethylsiloxane (PDMS), a silicone rubber that is biocompatible, optically transparent, flexible, and gas permeable [25,26]. These features have made this material an ideal choice for most CoC applications. Other materials, such as glass and thermoplastics, have also been used, but PDMS is particularly advantageous due to its mechanical resemblance to soft tissue and its versatility as a liquid polymer allowing for straightforward manufacturing through replica moulding [27]. However, the main limitation of PDMS is its hydrophobicity which leads to a tendency to absorb hydrophobic drug compounds during chemical screening [28]. An alternative to PDMS is poly (methyl methacrylate) (PMMA), a thermoplastic material less likely to absorb small, hydrophobic molecules [29].

Another method of manufacturing CoCs is by direct or indirect 3D printing [30]. Direct 3D printing involves printing the chambers and microchannels of the chip directly using a biocompatible material. Resins are commonly used for traditional direct 3D printing methods—stereolithography (SLA), digital light processing (DLP)—for their excellent printability, but many resin types have been found to be toxic [31]. To improve biocompatibility, resin-based chips can be coated with non-cytotoxic compounds, but it may be preferable to use inherently biocompatible resins or other materials like PDMS or PMMA. Guttridge et al. [32] published a comprehensive summary of commercially available biocompatible 3D printing resins.

Indirect 3D printing provides a faster and more cost-effective alternative to photolithography for creating moulds. Both non-sacrificial moulds (printed mould is used to cast the polymer) or sacrificial moulds (printed material is removed after polymer cross-linking to leave behind the desired structure) are possible [32]. This method is significantly faster than photolithography, enabling rapid prototyping and improving scalability, with the potential for mass production without the need for specialised clean-room facilities. Thus, this manufacturing strategy offers a highly promising approach for the future development of CoC models. It is important to note that SLA resins have been found to inhibit PDMS curing, and these 3D printed moulds need to be treated in various methods reviewed by Venzac et al. [33].

In addition to these approaches, 3D bioprinting using bioinks has recently emerged as a complementary strategy for organ- and tumour-on-chip fabrication. By depositing cell-laden hydrogels in defined patterns, bioprinting enables the reconstruction of tissue-like architectures and TME [17], although its application to ovarian cancer CoC models remains limited.

Photolithography, soft-lithography, and 3D printing each present distinct advantages and limitations in the fabrication of CoC platforms. Photolithography offers the highest resolution, routinely down to tens of nanometres, with high throughput suitable for large-scale production [34]. Liquid photolithography with SU-8 allows tunable feature thicknesses ranging from <1 µm up to ~500 µm, matching cellular microenvironmental scales, but requires cleanroom infrastructure, suffers from edge-bead effects, and may need toxic adhesion promoters [34]. Dry photolithography using laminated films such as ADEX reduces cost and processing time, provides excellent flatness and adhesion, and can be performed outside cleanrooms, although film thickness is fixed and multilayer patterning is less feasible [21,34]. In contrast, 3D printing generally achieves lower resolution (~1 µm for extrusion or inkjet-based approaches) but allows rapid prototyping, design flexibility, and cost-effectiveness, making it attractive for incremental chip development [34].

### 3.2. Review of CoC Design Approaches Used in Cancer Research

Either a bottom-up or top-down approach can be used to incorporate cellular components into CoC systems.

#### 3.2.1. Bottom-Up Approach

**Figure 2:**
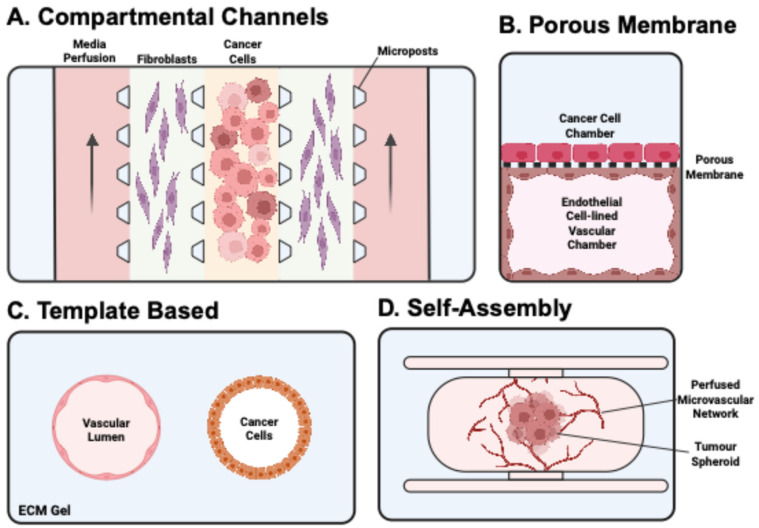
The four distinct bottom-up CoC design approaches for studying cancer metastasis, as identified by Jouybar et al. [35]. Created in BioRender. Lim, S. (2025) https://BioRender.com/pg8flzn, accessed on 20 August 2025.

A bottom-up, synthetic biology approach involves culturing isolated cells from primary, immortalised, or stem cell-derived sources within an initially empty microfluidic environment, allowing them to self-organise and remodel into functional tissue. This approach enables greater control over cell compartmentalisation, fluid routing, and spatial organisation within the CoC model. These designs can be further subdivided into four distinct categories identified by Jouybar et al. [35]: compartmentalised channels, porous membrane, template-based, and self-assembly (Figure 2).

Compartmentalised channel

The compartmentalised channel design consists of multiple parallel channels, with one channel often containing a hydrogel scaffold embedded with cancer or stromal cells, while adjacent channels are lined with endothelial cells. These endothelial cell-lined vascular channels are continuously perfused with media, serving as a microfluidic blood vessel. These platforms usually line microchannels with microposts to contain hydrogel within a channel but allow for free movement of cells and media. For example, this approach has been used to culture self-arranged endothelial cells [36], vascularise spheroids [37], and generate chemotactic gradients [38]. The ability to have a vascular channel adjacent to ECM hydrogels has been commonly used to study cancer cell invasion and extravasation dynamics [39]. These chips can also be perfused with therapeutics, providing a platform that can mimic in vivo drug pharmacokinetics or to study drugs targeting metastasis.

Porous membrane

The porous membrane design typically features a single microchannel divided into two compartments by a permeable membrane. This membrane, commonly made of polycarbonate, polyethylene terephthalate (PET), or PDMS, contains micropores smaller than 10 μm. Many designs incorporate a vascular channel lined with endothelial cells, while epithelial and cancer cells are cultured in the compartment above. The porous membrane facilitates cell migration, making it a valuable tool for modelling cancer cell stromal invasion [40] and intra/extravasation [41].

Template-based

In a template-based design, a hollow internal lumen is formed either by using a temporary mould or forcing fluid through an ECM gel [42]. This lumen can be lined with cells to model blood vessels or ducts. Cancer cells can then be seeded in the surrounding gel or perfused through the channel to study metastasis.

In general, template-based models require minimal microfabrication, making them straightforward and highly translatable. For example, Moon et al. [43] designed a simple model in which a lumen lined with breast cancer cells was used to study their invasion into a collagen gel. More complex designs are also possible, and Nguyen et al. [44] utilised this design to model pancreatic ductal adenocarcinoma (PDAC) interactions with blood vessels. Their model consisted of two parallel lumens (a pancreatic cancer duct and endothelial-lined perfused blood vessel), and the PDAC cells were observed to invade the gel towards the vessel lumen.

Self-assembly

The self-assembly design often involves seeding endothelial cells within a gel to allow for their self-assembly into a 3D vascular network around tumour cells. This design has similarities with the compartmentalised design but relies less on microchannel compartmentalisation of numerous cell types. For example, Boussommier-Calleja et al. [45] cultured a self-arranged vascular network within a fibrin gel embedded with fibroblasts. The endothelial cells formed lumens that connected to microfluidic channels through which media, monocytes, and tumour cells were perfused.

#### 3.2.2. Top-Down Approach

In a top-down (organotypic) approach, primary tissues, such as organ slices from patient biopsies, are directly integrated into the chip. These ex vivo samples retain native tissue architecture, morphology, cellular composition, extracellular matrix (ECM), and heterogeneity [19]. However, traditional “static” culture techniques for explants have struggled to maintain tissue viability, primarily due to the lack of vascular perfusion needed to deliver oxygen and nutrients while removing metabolic waste. Any reconstructed tissue or organ exceeding 400 µm is considered to need functional vascularisation [46]. Culturing these samples under continuous media flow has been shown to improve their viability [47]. Moreover, precision-cut tissue (PCT) slices, where explants are cut into sub-millimetre thin slices, have been observed to enhance nutrient diffusion [48]. While these advancements have helped mitigate the inevitable decline in metabolic activity over time, CoC technology offers even greater potential by precisely controlling fluid flow and tissue orientation.

#### 3.2.3. Media Perifusion

Designs have been implemented to facilitate the constant “perifusion” of media around a tissue slice. van Midwoud et al. [49] used polycarbonate membranes above and below a liver PCT slice (100 µm) to secure the slice as media were perpendicularly perifused from top to bottom within a teardrop-shaped chamber. The shape of the chamber and the membranes ensured even distribution of flow across the tissue slice and the system. This flow meant the media were refreshed within the chamber every 2.5 min, but viability was still comparable to standard well culture. In a different approach, Kennedy et al. [50] utilised sintered discs to mimic microvascular diffusion, maintaining head and neck cancer tissue viability for 68 h, significantly longer than typical static cultures.

A key challenge in these perifusion designs is securing the tissue under media flow while ensuring compatibility with imaging and easy access to the tissue throughout the culture period. Amirabadi et al. [51] developed a chip that secures intestinal explants with a removable cap. This design featured an innovative cantilever system with “snap-fit” hooks that clicked the cap into place, ensuring media perfusion above and below the tissue within a sealed yet accessible chamber.

#### 3.2.4. Drug Screening Platforms

A different approach for utilising tumour slices is through high-throughput drug screening platforms, such as the microfluidic device created by Horowitz et al. [52]. This chip incorporates 40 parallel microchannels that deliver different drugs simultaneously across a single tumour tissue section, enabling multiplexed testing of chemotherapy agents and drug combinations.

### 3.3. Review of CoC Models Used in Ovarian Cancer Research

**Figure 3:**
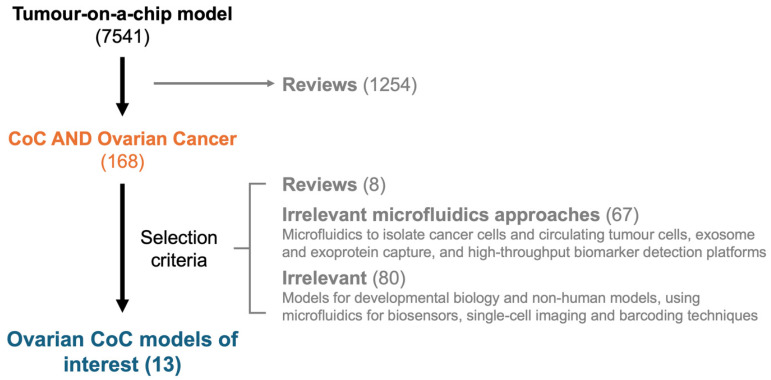
Results of the literature search conducted to identify CoC models in ovarian cancer.

A PubMed search combining the keywords for CoC models and OC yielded 168 papers (Figure 3). Of these, 8 reviews and 80 studies on developmental biology, non-human models, biosensor development, and unrelated microfluidic techniques were excluded. Additional 66 papers were removed as they focused on analytical and diagnostic applications, such as exosome-capturing chips, high-throughput biomarker detection platforms, and microfluidic systems for isolating OC cells or performing single-cell analyses. Ultimately, 13 papers that modelled the ovarian TME were selected. A detailed summary of these papers is provided in Table 1.

#### 3.3.1. Simple Designs to Introduce Biomechanical Forces

Simple CoC designs can introduce physiologically relevant mechanical stimuli to OC cells. Rizvi et al. [53] cultured 3D OC micronodules under media flow within a simple Matrigel-lined microchannel system. Continuous fluid shear stress not only reduced tumour volume compared with static cultures but also induced morphological, genetic, and protein changes consistent with epithelial–mesenchymal transition (EMT). Specifically, the flow down-regulated E-cadherin, up-regulated vimentin, and activated EGFR signalling, driving a more motile and aggressive tumour phenotype. Li et al. [54] outlined a protocol for perfusing OC spheroids over a peritoneal mesothelial layer, simulating tumour cell aggregates migrating via peritoneal fluid to the omentum. This dynamic 3D OC–mesothelium platform emphasised the role of shear stress in promoting tumour–mesothelial interactions during peritoneal dissemination, providing a biologically relevant model of early metastatic colonisation. Both systems used pressure-driven media flow within a microchannel to exert biomechanical forces on OC cells.

#### 3.3.2. Compartmentalised Designs

Most studies employed a compartmentalised design. Hwang et al. [55] developed an on-chip migration assay, tracking individual SKOV-3 cells embedded in collagen as they moved along a chemotactic gradient. Likewise, Scott et al. [56] modelled macrophage infiltration towards embedded OC cells using a similar design. This study showed that tumour-derived CSF1 drives density-dependent macrophage recruitment, which is further amplified by chemotherapy, consistent with patient-derived xenograft profiles.

However, the multichannel feature of the compartmentalised design has mainly been used to model the vasculature. Wimalachandra et al. [57] incorporated two endothelial channels lined with human umbilical vein endothelial cells (HUVECs) around a central tumour chamber, creating a tumour–vascular interface. Using this system, they confirmed that folic acid- and chemokine-loaded nanoparticles could cross the endothelial barrier, target folate receptor-positive OC cells, and simultaneously attract dendritic cells and T cells, highlighting the potential of nanoparticle-mediated immunotherapy. Buckley et al. [58] expanded on this by allowing endothelial and fibroblast cells to self-assemble into a vascular network. Daily media refresh from the vascular chamber induced physiologically relevant interstitial flow in adjacent OC chambers. Under these conditions, OC cells experienced tensile strain and upregulated HSP27, implicating a mechanobiological route to chemoresistance.

Joy et al. [59] also followed the self-assembling principle to vascularise an OC tumour spheroid. Fibroblasts and endothelial cells were seeded onto an ECM gel, and over 7 days, they formed cancer-associated fibroblasts (CAFs) and a functional vasculature around the tumour spheroid. This vascularised model enabled chimeric antigen receptor (CAR) T-cell perfusion, showing that fibroblasts can either augment CAR T activity through chemokine signalling or restrict it via ECM-driven TGF-β pathways, thus uncovering mechanisms of immune evasion. Furthermore, they were able to collect the effluent of the microfluidic chip to measure levels of proinflammatory cytokines [59].

#### 3.3.3. Complex Multi-Compartment Designs

The compartmentalised approach has also enabled more complex models incorporating additional TME components. Plesselova et al. [60] developed a chip to model the complex gradients and stromal interactions within the ovarian TME. The device features a central OC chamber flanked by two stromal compartments containing endothelial cells co-cultured with either normal fibroblasts or CAFs. The two outermost circulation channels are perfused with media and drugs, generating fluid shear stress and nutrient gradients. The inclusion of these multiple compartments allowed for the modelling of numerous aspects of OC, such as metastatic invasion, CAF-mediated ECM remodelling, hypoxic gradients, and drug resistance.

Another innovative approach using the compartmentalised design was developed by Ibrahim et al. [61] to model OC omental metastasis. Their device featured a mesothelial monolayer and an underlying adipocyte-rich stroma within a perfusable microvascular network. OC cells were introduced on top of the mesothelial layer to recapitulate key interactions such as tumour cell adhesion, stromal cell-driven vascular remodelling, and mesothelial barrier permeability. The study revealed a tumour cell density threshold required for sustained growth, which was further enhanced by adipocytes and endothelial cells, while tumour expansion altered microvascular permeability through both mechanical compression and cytokine signalling—mechanisms relevant to ascites formation [61]. This chip was the first in vitro vascularised model of the peritoneum and the omental TME.

#### 3.3.4. Porous Membrane Designs

Several studies incorporated the porous membrane design to model key TME interactions. Surendran et al. [62] used a porous membrane to perfuse neutrophils above OC spheroids seeded in a hydrogel to create an accurate in vitro tumour-immune microenvironment (TIME). Neutrophils were seen to navigate through the collagen matrix towards OC spheroids through chemotaxis and neutrophil extracellular trap (NET) production.

In a different approach, Saha et al. [63] developed the tumour–vessel–blood-integrated organ chip (OvCa-Chip) to model the tumour–vessel–blood interface and platelet extravasation. The chip consisted of a perfused vessel chamber and a tumour chamber separated via a matrix-coated porous membrane. Platelets were perfused over 5 days, and their adhesion to the tumour-associated endothelial cells and extravasation into the tumour chamber was observed. The OTME-Chip uncovered a shear-dependent mechanism in which platelet glycoprotein VI (GPVI) engages tumour galectin-3, promoting invasion and chemo-resistance. Saha et al. [65] expanded the OvCa-Chip into the ovarian tumour microenvironment organ-on-chip (OTME-Chip). By analysing effluents from the tumour and endothelial chambers, the authors identified cytokine and transcriptional programmes through which cancer-associated endothelium loses barrier integrity, thereby promoting platelet adhesion and extravasation. The OTME-Chip also added two flanking hydrogel ECM channels, enabling quantitative assessment of OC cell invasion and permitting direct comparisons across conditions.

**Figure 4:**
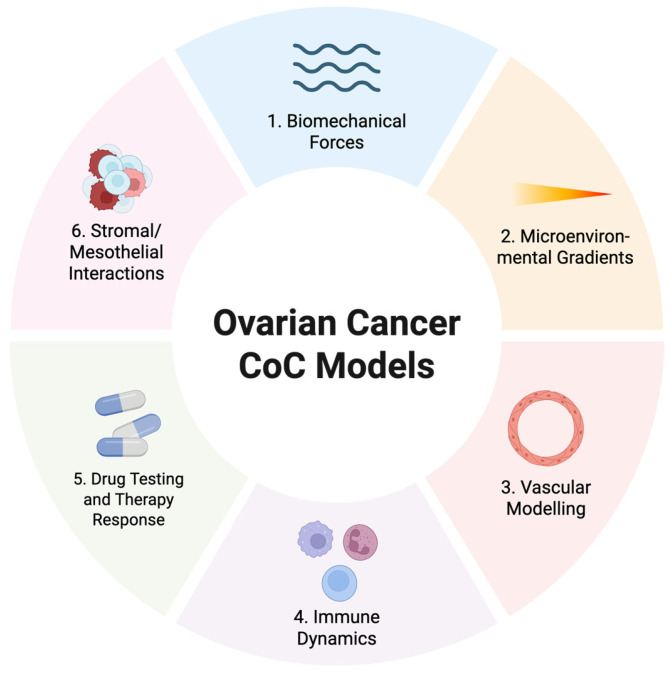
Broad experimental outcomes demonstrated by OC CoC models. Published OC-specific CoC platforms have been applied across six major domains: (**1**). Biomechanical forces—fluid shear stress and interstitial flow induce EMT, stemness changes [53], and invasion dynamics [55]; (**2**). Microenvironmental gradients—generation of nutrient and oxygen gradients, hypoxia-driven phenotypes, and heterogeneous drug penetration [60]; (**3**). Vascular modelling—endothelial dysfunction, platelet extravasation [63,64], and nanoparticle transport across vessel–tumour interfaces [57]; (**4**). Immune dynamics—macrophage infiltration via CSF1 signalling [56], neutrophil chemotaxis and NET formation [62], and CAR-T cell extravasation [59]; (**5**). Drug testing and therapy response, on-chip chemoresistance [57,62,63,64]; (**6**). Stromal and mesothelial interactions—fibroblast- and CAF-mediated matrix remodelling [60], adipocyte-induced vascular and mesothelial permeability, and tumour adhesion to the mesothelium [54,61]. Together, these outcomes highlight the versatility of CoC technology in examining diverse aspects of OC biology and its translational potential for therapeutic testing. Created in BioRender. Lim, S. (2025) https://BioRender.com/rwva7uw, accessed on 20 August 2025.

Importantly, these OC COC studies collectively highlight distinct biological insights that can be grouped into six major themes: biomechanical forces, microenvironmental gradients, vascular modelling, immune dynamics, drug response, and stromal/mesothelial interactions (Figure 4).

### 3.4. Extracellular Matrix–Specific Effects on Ovarian Cancer Biology

Across OC CoC models, the ECM is not just a scaffold but a key driver of tumour behaviour. Matrigel (basement membrane-rich) beds supported adhesion and 3D micronodule formation under flow and, critically, coupled with shear stress to induce EMT-like programs and reduce proliferation, indicating a flow–ECM synergy that promotes motility and aggressiveness [53]. Fibronectin-coated mesothelium enabled spheroid–mesothelium adhesion/clearance assays under physiologic shear, capturing early peritoneal attachment dynamics and providing a tractable surface to test anti-adhesion strategies [54]. In vascularized chips, tuning a fibrin–collagen-I composite altered microvessel architecture (coverage, lacunarity), thereby reshaping interstitial transport and the microenvironment experienced by OC cells; under these conditions, tensile strain modulated HSP27 signalling and apoptosis markers, linking ECM composition/mechanics to chemoresistance phenotypes [58]. A human plasma-derived fibrin matrix with stromal co-cultures revealed CAF-driven ECM remodelling, oxygen/drug penetration gradients, and CAF-modulated drug response, directly tying stromal–ECM reprogramming to resistance [60]. Finally, collagen-I hydrogels used as invasion compartments in the OTME-Chip enabled real-time quantification of platelet-triggered invasion/proliferation and uncovered a shear-dependent pathway driving invasion and chemoresistance, testing its blockade and demonstrating ECM-anchored, mechanism-guided therapeutic testing in chip [63]. Together, these studies show that ECM identity (Matrigel, fibrin, collagen, plasma-derived) and architecture/mechanics decisively influence adhesion, invasion, proliferation, transport/penetration, and drug response in OC CoC platforms [53,54,58,60,61,63]. Although direct head-to-head comparisons of different ECMs in ovarian cancer CoC systems are lacking, the collective evidence underscores ECM as a critical determinant of tumour behaviour that warrants more systematic investigation in future models.

### 3.5. Points to Be Considered When Approaching New Designs for CoC Models

**Figure 5:**
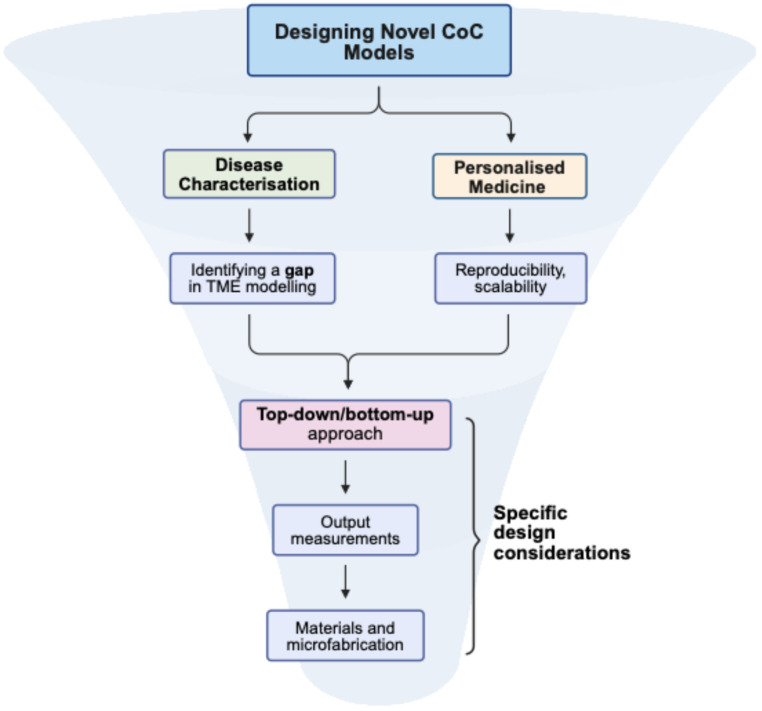
A systematic approach for designing novel CoC models, with increasingly specific design considerations closer to the bottom of the figure. Created in BioRender. Lim, S. (2025) https://BioRender.com/kn3qyke, accessed on 20 August 2025.

Understanding current CoC technology, observing design trends, and critically evaluating the strengths and weaknesses of published models can guide the development of robust and relevant CoC platforms for ovarian cancer research (Figure 5).

When designing a CoC model, it is important to distinguish between platforms intended for fundamental disease characterisation and those aimed at clinical applications in personalised medicine. Disease-characterisation models should address specific gaps in current TME modelling strategies, with clear justification for the use of microfluidics and specific CoC features. For personalised medicine, key design goals include reproducibility, scalability, rapid data acquisition, user-friendliness, and ease of manufacture. Moreover, clearly defined outcome measures for drug response or disease progression are essential to facilitate clinical interpretation.

A central design decision is whether to adopt a bottom-up or top-down approach. Top-down tissue-culture chips are often limited by shorter culture periods, but they preserve patient-specific tumour and TME heterogeneity, making them particularly valuable for personalised medicine applications [61]. Conversely, bottom-up strategies enable precise assembly of selected cell types, ECM components, and microfluidic features, allowing detailed study of cancer processes such as stromal interactions, metastasis, and drug resistance under controlled gradients and flow conditions [55,57]. This approach also provides flexibility in selecting an appropriate design subtype—or hybrid combinations—to best match the biological question.

Equally important, outcome measurements should guide material and design choices. For example, studies prioritising real-time imaging require optically transparent substrates such as PDMS or glass to avoid obstructed views [26], while biochemical assays may demand designs optimised for media collection. Aligning these technical requirements with overall design decisions will improve reproducibility and translational value.

## 4. Discussion

To our knowledge, this is the first comprehensive review examining current advancements in CoC platforms for OC modelling, with a focus on design diversity, fabrication methods, and TME integration. This article is uniquely dedicated to ovarian cancer and systematically integrates not only engineering advances but also biological findings from existing models. By categorising design strategies—ranging from photolithography-based compartmentalised systems to emerging 3D-printed platforms—and systematically analysing OC-specific implementations, this review aimed to clarify the existing landscape and identify gaps in biological relevance and technological consistency. In addition, by outlining structured design considerations in Section 3.4, we provide a forward-looking framework to guide the rational development of next-generation OC CoC platforms with translational and personalized medicine potential.

A unique strength of CoCs is their ability to host multiple, spatially segregated compartments with distinct cell types to study several disease processes. Therefore, Ibrahim et al. engineered an omentum-on-chip incorporating vascular endothelium, mesothelium, adipocytes, and stromal elements to model early attachment, growth, and permeability changes during intraperitoneal metastasis [61]. While this platform necessarily simplified features of the omentum (e.g., unrepresentative cell-line choices and stromal composition), it illustrated the feasibility of assembling numerous TME components in one device. Building on these advances, there is a clear opportunity to couple ascites-like flow with mesothelial clearance and omental invasion in OC-specific CoCs, especially with the development of a simple peritoneal microdevice by Li et al. [53] that allows for the co-culture of the mesothelium with OC spheroids under flow.

Despite its strengths, several critical limitations must be addressed before they can be widely adopted in translational and personalised medicine. The following sections outline the primary biological, engineering, and standardisation challenges, and highlight potential directions for future work in OC research.

### 4.1. Biological Challenges

Most current OC CoC models rely on established cell lines such as SKOV-3, OVCAR-3, and A2780 [53,54,55,56,63], which lack the genomic complexity of high-grade serous carcinoma (HGSC), the most prevalent and aggressive OC subtype [2,3]. Only a few models incorporate patient-derived xenograft (PDX) cells or primary cells [56,59], and multi-type co-culture systems remain technically challenging due to differing growth requirements.

Efforts to mimic the TME remain incomplete. While several models include mesothelial [54,61], endothelial [58,59,61], stromal [60,61], or immune cells [56,62], very few integrate more than two TME elements. Adipocytes—despite their essential role in omental metastasis—are represented in only one model [61]. Immune–tumour interaction modelling is also underdeveloped. Joy et al. [59] integrated CAR-T cells in a vascularised chip, but broader incorporation of adaptive immunity remains rare. Emerging approaches now demonstrate that OC CoC platforms can be leveraged to co-culture tumour cells with patient-derived immune cells [59], or to introduce chemokine gradients such as CCL21 to direct lymphocyte and dendritic cell recruitment [57], while TIME-on-Chip systems recreate neutrophil trafficking and NETosis [62]. These strategies illustrate the feasibility of modelling complex immune–tumour dynamics and point to promising directions for future OC CoC development, which are further elaborated in Section 4.4.

Ascites is a defining feature of advanced OC and plays a multifactorial role in disease progression [53,61]. The ascitic fluid contains high levels of cytokines and growth factors such as IL-6, IL-8, VEGF, and TGF-β, along with extracellular vesicles and bioactive lipids, which collectively promote tumour proliferation, EMT, angiogenesis, and immune suppression [9]. The mechanical accumulation of ascites also generates hydrostatic pressure that facilitates tumour cell detachment from the primary site and enhances peritoneal dissemination [9]. Furthermore, the acellular and cellular fractions of ascites—including mesothelial cells, fibroblasts, and immune cells—create a permissive niche that supports chemoresistance and recurrence [53,61].

A limited number of CoC platforms have attempted to model aspects of this biology. Microfluidic peritoneal devices seeded with OC spheroids embedded in Matrigel under continuous perfusion flow demonstrated how sustained shear stress reduces nodule size yet induces EMT-like changes, with downregulated E-cadherin and upregulated vimentin mimicking the mechanical effects of ascitic fluid movement [53]. In another approach, a vascularized omental microtissue-on-chip incorporating endothelial cells, adipocytes, and mesothelial layers in a fibrin matrix recapitulated stromal remodelling of the peritoneal barrier, revealing how adipocyte–mesothelial crosstalk alters vascular permeability, a mechanism linked to ascites accumulation [61]. More recently, microdevices have also used hydrogel matrices or suspension culture systems to simulate the buoyant environment of tumour spheroids in ascitic fluid, enabling drug testing under conditions that better resemble the ascitic niche [54].

Despite these advances, no OC CoC platform has yet integrated the full biochemical complexity of patient-derived ascites or the hydrostatic pressures observed in vivo, leaving ascites biology incompletely recapitulated in current systems.

### 4.2. Technical Limitations

Polydimethylsiloxane (PDMS) remains the dominant material for microfluidic chip fabrication due to its optical clarity and biocompatibility [25,26]. However, PDMS absorbs hydrophobic drugs such as paclitaxel, limiting its use in pharmacological studies [28]. Alternatives like PMMA [29] or coated PDMS surfaces show promise but are underutilised.

Fabrication techniques present another challenge. Photolithography, while precise, requires specialised clean-room facilities. Indirect 3D printing offers faster, scalable alternatives [31], yet issues with resin toxicity [31] and PDMS curing inhibition [33] persist. Using alternatives as indirect 3D printing and biocompatible resins can reduce reliance on expensive cleanroom facilities, thereby lowering manufacturing costs. None of the reviewed OC CoC models have yet used 3D printing as the primary manufacturing approach.

Recent advances are beginning to address these limitations. A growing number of inherently biocompatible photosensitive resins are now commercially available, with certifications under ISO 10993 or USP Class VI, enabling safer use in biomedical and microfluidic applications [32]. In parallel, surface treatments of 3D-printed moulds, including fluorinated silane coatings and omniphobic lubricant-infused modifications, have been shown to prevent PDMS curing inhibition and improve optical quality, making them suitable for repeated long-term casting [30]. Moreover, photocurable PDMS formulations have been developed for direct vat-photopolymerisation printing, yielding transparent elastomeric devices, while sacrificial and embedded 3D printing strategies enable the fabrication of complex, perfusable networks using biocompatible hydrogels or PDMS [30]. Although no OC CoC models to date have implemented these emerging solutions, their adoption could markedly improve the accessibility, reproducibility, and translational potential of OC-specific microfluidic systems.

### 4.3. Lack of Standardisation

There is no consensus on protocols for chip design, flow rates, ECM composition, or readouts. ECMs used in OC chips vary widely—from Matrigel [53] and collagen [55] to patient-derived fibrin matrices [60]—each affecting cell behaviour differently. Output measures also differ substantially, ranging from fluorescence microscopy to RNA sequencing and cytokine profiling [58,64], making cross-study comparisons difficult.

Both tumour spheroids and the mesothelial layer experience continuous fluid shear under perfusion. However, the physiological wall shear stress (WSS) within the peritoneal cavity is not yet precisely defined and is likely heterogeneous in space and time (e.g., respiration, peristalsis, posture, and ascites volume). Computational estimates span ~0.14–11 dyn·cm^−2^ [66,67], and even very low shear can modulate OC phenotypes—Ip et al. [68] reported stemness changes at ~0.1 dyn·cm^−2^. Thus, studies should report both imposed flow rates and calculated WSS to help place results into context, improve cross-study comparability, and converge on a benchmark WSS.

### 4.4. Future Prospects

Several strategies could enhance the biological relevance and scalability of CoC models in OC research:

Patient-derived materials: Using autologous tumour cells and patient-derived ECM can improve model fidelity [69].

Expanded immune modelling: Inclusion of T cells, dendritic cells, and NK cells would enable more accurate study of tumour–immune dynamics, as shown by the CAR-T example in Joy et al. [59]. In particular, the systematic integration of adaptive immune components represents a critical next step. Incorporating patient-derived T cells, dendritic cells, or NK cells into OC CoC systems [59], together with engineered cytokine gradients to direct immune cell recruitment [57], could enable physiologically relevant modelling of tumour–immune dynamics. Similarly, TIME-on-Chip approaches that capture neutrophil trafficking and NETosis [62] provide complementary strategies for recapitulating innate immune functions. Such immune-inclusive designs, especially when combined with patient-derived tumour materials, hold strong potential to advance personalised medicine and support the preclinical development of immunotherapies in ovarian cancer.

Ascites-on-chip platforms: Current OC CoC models largely replicate shear stress but do not capture the biochemical complexity or hydrostatic pressure of malignant ascites. Future systems could integrate pressure control, patient-derived ascitic fluid, and stromal or immune compartments to more faithfully model ascites biology and its impact on invasion, immune suppression, angiogenesis, and drug resistance.

Multiplexed drug testing: Systems like the 40-channel platform by Horowitz et al. [52] allow high-throughput testing and could be adapted to OC samples.

Live imaging and biosensing: Integrating optical access and biosensors enables real-time monitoring of invasion, signalling, and metabolic changes.

Open-access design and reporting standards: Sharing CAD files, ECM formulations, and standardised analytical methods would improve reproducibility. At a minimum, authors should disclose (i) wall shear stress or equivalent flow parameters, given their known role in modulating OC cell phenotype [53], (ii) ECM composition and source, which directly influence adhesion, invasion, and therapy response [53,54,61,63], (iii) the origin of tumour and stromal cells, ideally distinguishing between cell lines and patient-derived materials [59,61], and (iv) key functional readouts such as proliferation, invasion, EMT, immune interactions, or drug response. Consistent reporting of these parameters would enable cross-study comparisons and support systematic development of OC CoC platforms.

### 4.5. Clinical Translation

For clinical adoption, CoC platforms must be straightforward to operate, scalable, and compatible with patient-derived samples. Designs that integrate relevant TME elements, physiological flow, and accessible analytical outputs offer a strong foundation for translational use. Incorporating metabolic components such as adipocyte signalling and validating results against patient data will be essential steps toward establishing CoC systems as reliable tools for personalised medicine and drug development.

## 5. Conclusions

Tumour-on-chip platforms represent a powerful step forward in modelling ovarian cancer, offering unique opportunities to capture the complexity of the TME and overcome the limitations of conventional in vitro and in vivo systems. Although current studies remain limited in number and lack standardisation, early applications demonstrate their potential to reproduce key biological processes such as metastasis, immune interactions, and drug responses. To fully unlock this potential, future efforts must prioritise the use of patient-derived materials, integration of diverse stromal and immune components, adoption of scalable manufacturing methods, and establishment of shared design and reporting standards. By addressing these challenges, next-generation tumour-on-chip models could accelerate discoveries in ovarian cancer biology, enable reliable preclinical drug testing, and ultimately pave the way for personalised therapeutic strategies with greater clinical relevance.

## Figures and Tables

**Table 1:** Published tumour-on-chip models applied to ovarian cancer research.

Device	Objective	Design Approach	TME Cells Used	OC Cells	Matrix	Output Measurements	Main Outcome
OC micronodules in microfluidic platform [53]	Investigate the role of fluidic forces on metastasis	Microchannel	N/A	OVCAR-5	Growth-factor reduced (GFR) matrigel	Fluorescent microscopy, RT-PCR, WB	OC cells under flow had morphological, genetic, and protein profiles associated with increased epithelial–mesenchymal transition
Multicellular Spheroid in Peritoneal Microdevice [54]	OC spheroids perfused over a layer of mesothelial cells	Microchannel	Peritoneal mesothelial cells	SKOV-3	Fibronectin coating	N/A	OC spheroids were successfully cultured over mesothelial cells under flow
Microfluidic Chemotaxis assay [55]	Tracking the chemotaxis of individual cells and response to RhoA/ROCK inhibition	Compartmentalised channel	N/A	SKOV-3	Collagen gel and agarose	Fluorescent microscopy, image-based cell tracking assay	SKOV-3 migration speed and directional persistence measured, RhoA/ROCK pathway inhibition reduced directional persistence
Macrophage Migration Dynamics in a 3D Microfluidic Model [56]	Modelling macrophage infiltration towards OC cells embedded in ECM matrix	Compartmentalised channel	Macrophages	ID8, DF83 and DF216 patient-derived xenografts	Collagen-1 gel	Fluorescent microscopy, enzyme-linked immunosorbent assay (ELISA)	Macrophage recruited in response to both direct and paracrine interactions with OC cells, CSF1 signalling important for macrophage-rich OC TIME
Simulated Blood Vessel–Tumor System [57]	Testing for folic acid-conjugated nanoparticle’s ability to target tumour cells and recruit dendritic and T cells within a vasculature–tumor interface	Compartmentalised channel	Endothelial, Jurkat, dendritic cells	OVCAR-3	Fibrinogen/thrombin gel, fibronectin coated channel	Fluorescent microscopy	Nanoparticle uptake across the endothelial barrier was verified and T and dendritic cells were recruited.
Vascularised TME model [58]	Observing changes to mechanically stimulated cells when they are cultured in a new environment	Compartmentalised channel	Endothelial cells	SKOV-3, SKOV-3 taxol resistant, OVCAR-8	Fibrinogen and collagen-1 gel	Immunofluorescence, Western blot (WB)	When strained cells were incorporated into the chip, they demonstrated mechanical memory by maintaining their heat shock protein (HSP) expression
Vascularised spheroid microfluidic chip [59]	Modelling CAR T-cell activity in a vascularised spheroid chip	Compartmentalised channel	Endothelial cells, fibroblasts	G164, G33	Fibrin and collagen gel	Fluorescent microscopy, immunofluorescence, chip effluent collected for cytokine assay	CAR T-cells extravasated vascular network, penetrated ECM gel, and induced apoptosis in OC cells
Multi-vascularised multi-niche tumour-on-chip [60]	Generating nutrient and oxygen gradients, stromal interactions, drug uptake	Compartmentalised channel	Normal fibroblasts, CAFs, endothelial cells	KURAMOCHI, SKOV-3	Human plasma matrix based on fibrinogen crosslinking	Fluorescent microscopy, immunofluorescence, imaging flow cytometry	Tumour-CAF interactions, invasion into stromal compartments, CAF induced ECM remodelling, hypoxia, drug resistance
Omentum-on-a-chip [61]	Modelling the omental TME and ascites formation	Compartmentalised channel	Adipocytes, endothelial and mesothelial cells	SKOV-3, OV90, OCVAR3	Fibrin hydrogel	Permeability assays using fluorescent tracers, fluorescent microscopy	Adipocytes increase mesothelium and vessel permeability, ECM composition, and mesothelium vulnerability to tumour attachment
Tumor-immune microenvironment (TIME)-on-Chip [62]	Modelling neutrophil recruitment and activation and OC invasion	Porous membrane	Neutrophils	OVCAR-3	Collagen gel and hydrogel microwells	Immunostaining	Neutrophils respond to tumour spheroid by chemotaxis and neutrophil extracellular traps.
OvCa/OTME-chip [63,64]	OC interaction with platelets, metastasis	Porous membrane	Endothelial cells	A2780	Collagen–fibronectin-coated porous PDMS membrane, collagen-1 hydrogel	Fluorescent microscopy, chip effluent collected for cytokine assay, flow cytometry, RNAseq	OC cells induced vascular dysfunction, demonstrated platelet extravasation and tumour interactions
Mutlilayered cancer-on-a-chip model [65]	Multilayered co-culture to study effectiveness of photodynamic therapy	Microchamber	Fibroblasts	A2780	N/A	Fluorescent intensity quantification using plate-reader, fluorescence microscopy.	Novel photosensitisers’ effectiveness was verified

## Data Availability

The datasets used and/or analysed during the current study are available in the list of references cited.
